# Indications and Mechanisms of Action of the Main Treatment Modalities for Non-Melanoma Skin Cancer

**DOI:** 10.3390/life15091447

**Published:** 2025-09-16

**Authors:** Marcio F. Chedid, Aline C. Tregnago, Floriano Riva, Lucas Prediger, Anisha Agarwal, Jane Mattei

**Affiliations:** 1 Postgraduate Program in Medicine, Surgical Sciences and Oncological and Transplantation Surgery, Hospital de Clinicas de Porto Alegre, Federal University of Rio Grande do Sul (UFRGS), Porto Alegre 90035-003, Brazil; 2 Brazilian Research Council (CNPQ), Brasilia 70070-010, Brazil; 3 Department of Pathology, Medical School, University of Caxias do Sul (UCS), Caxias do Sul 95070-560, Brazil; aline@cpmlab.com; 4 CPM Pathology Laboratory, Caxias do Sul 95020-170, Brazil; florianoriva@gmail.com; 5 Oncological and Transplantation Surgery, Hospital de Clinicas de Porto Alegre, Federal University of Rio Grande do Sul (UFRGS), Porto Alegre 90035-903, Brazil; lprediger@hcpa.edu.br; 6 Internal Medicine, Ascension Saint Joseph Hospital, Chicago, IL 60657, USA; anishaagarwal446@gmail.com; 7 Melanoma Medical Oncology, University of Texas in San Antonio, San Antonio, TX 78249, USA; mattei@uthscsa.edu

**Keywords:** skin cancer, non-melanoma skin cancer, surgery, immunotherapy, cemiplimab, pembrolizumab, chemotherapy, radiotherapy, micrographic Mohs surgery

## Abstract

Skin cancer is the most common cancer worldwide. The incidence of skin cancer has been increasing worldwide. Nearly 75% of all skin cancers are basal cell carcinomas (BCC), cutaneous squamous cell carcinoma (cSCC) represents approximately 20%, and those remaining are melanomas (4%) or other rare tumors (1%). Given the high cure rates and the ability to histologically confirm tumor clearance, surgical therapy is the gold standard for the treatment of skin cancer. Conventional surgery is the most employed technique for the removal of non-melanoma skin cancer (NMSCs). Mohs Micrographic Surgery (MMS) is the most precise surgical method for the treatment of non-melanoma skin cancer, allowing for 100% margin evaluation, being the gold-standard method for surgical treatment of non-melanoma skin cancer. Whenever it is possible to obtain wide margins (4 to 6 mm), cure rates vary from 70% to 99%. Imiquimod, a synthetic imidazoquinolinone amine, is a topical immune response modifier approved by the U.S. Food and Drug Administration (FDA) for the treatment of external anogenital warts, actinic keratosis (AK), and superficial basal cell carcinoma (sBCC). The efficacy of imiquimod is primarily attributed to its ability to modulate both innate and adaptive immune responses, as well as its direct effects on cancer cells. Imiquimod exerts its immunomodulatory effects by activating Toll-like receptors 7 and 8 (TLR7/8) on various immune cells, including dendritic cells, macrophages, and natural killer (NK) cells. Upon binding to these receptors, imiquimod triggers the MyD88-dependent signaling pathway, leading to the activation of nuclear factor kappa B (NF-κB) and interferon regulatory factors (IRFs). This cascade leads to the production of pro-inflammatory cytokines, including interferon-alpha (IFN-α), tumor necrosis factor-alpha (TNF-α), interleukin-12 (IL-12), and interleukin-6 (IL-6). These cytokines enhance local inflammation, recruit additional immune cells to the tumor site, and stimulate antigen presentation, thereby promoting an anti-tumor immune response. Radiation therapy (RTh) may be employed as a primary treatment to BCC. It may also be employed as an adjuvant treatment to surgery for SCC and aggressive subtypes of BCC. RTh triggers both direct and indirect DNA damage on cancer cells and generates reactive oxygen species (ROS) within cells. ROS trigger oxidative damage to DNA, proteins, and lipids, exacerbating the cellular stress and contributing to tumor cell death. Recently, immunotherapy emerged as a revolutionary treatment for all stages of SCC. Cemiplimab is a human programmed cell death 1 (PD-1)-blocking antibody that triggers a response to over 50% of patients with locally advanced and metastatic SCC. A randomized clinical trial (RCT) published in 2022 revealed that cemiplimab was highly effective in the neoadjuvant treatment of large SCCs. The drug promoted a significant tumor size decrease, enabling organ-sparing operations and a much better cosmetic effect. A few months ago, a RCT of cemiplimab on adjuvant therapy for locally aggressive SCC was published. Interestingly, cemiplimab was administered to patients with local or regional cutaneous squamous cell carcinoma after surgical resection and postoperative radiotherapy, at high risk for recurrence owing to nodal features, revealed that cemiplimab led to much lower risks both of locoregional recurrence and distant recurrence.

## 1. Introduction

Skin cancer is the most common cancer worldwide. Furthermore, the total number of new cases (incidence) of skin cancer has been increasing worldwide. From 2006 to 2012, the incidence of non-melanoma skin cancers (NMSCs) rose 35% in the Medicare population in the United States [1].

Prevention is much better than a cure. In this context, exposure to sunlight between 10 a.m. and 4 p.m. should be avoided, both in summer and winter. Even on cloudy days, radiation from the sun’s rays can cause serious damage to the skin and trigger skin cancer. Therefore, whenever outdoor exposure between 10 a.m. and 4 p.m. is necessary, it is recommended to use sunscreen, ideally with a protection factor of at least 30 for all of us. Even people with a dark complexion can be affected by skin cancer.

Self-care is the key to a long and healthy life. Self-examination is essential: our skin deserves the most careful attention. New lesions and skin spots, that we have doubts about, should be shown to a dermatologist. Consultation with dermatologists once a year helps greatly in preventing skin cancer. The dermatologist examines the skin of the patient’s entire body and directs biopsies of suspected lesions to be performed by a dermatologist or by a surgeon (oncologic or plastic). This is a narrative review on the main treatment modalities for non-melanoma skin cancer.

## 2. Epidemiology

Nearly 75% of all skin cancers are basal cell carcinomas (BCC), whereas cutaneous squamous cell carcinoma (SCC) represents approximately 20%, and the remaining are melanomas (4%) or other rare tumors [1,2]. The most common and least aggressive types of skin cancer are BCC and SCC: they are grouped into NMSCs. In the vast majority of cases, NMSCs are restricted to the skin, as BCC and SCC very rarely metastasize to other organs.

For NMSCs restricted to the skin, removal of the lesion along with a skin safety margin is the only treatment recommended. Given the high cure rates and the ability to histologically confirm tumor clearance, surgical therapy is the gold standard for the treatment of non-melanoma skin cancer [3]. When performed by a competent surgeon or dermatologist without delay, skin cancer removal surgery will result in a cure in the vast majority of cases, as usually the lesions are localized and at an early stage. In these cases, chemotherapy, radiotherapy or immunotherapy are not required.

For a primary (non-recurrent) BCC, complete removal of the tumor with both sufficient circumferential and deep margins negative for cancer yields a 99% chance of cure [4]. Most specialists agree that margins of 2 to 5 mm are sufficient to achieve a good cure rate [5].

SCCs are more aggressive NMSCs than BCC. SCCs usually warrant wider margins than those of BCCs. The goal margins to enable chances of cure of 95% for SCCs range from 5 mm (for less aggressive forms of SCCs) to 13 mm (for aggressive forms of SCCs, involving the recurrent and ulcerated ones) [6].

NMSCs are associated with perturbations of redox homeostasis and decreased antioxidant activity. The microenvironment of NMSC patients exhibits lower levels of non-enzymatic antioxidants and increased byproducts of lipid, protein, and DNA oxidative damage. In these patients, pro-inflammatory interleukins are increased, anti-tumor biomolecule levels are reduced, and both immune response markers, as well as elevated vitamin D levels, are enhanced [7].

## 3. Mechanisms of Action of Imiquimod in the Treatment of Basal Cell Carcinoma and Actinic Keratosis

### 3.1. Introduction

Imiquimod, a synthetic imidazoquinolinone amine, is a topical immune response modifier approved by the U.S. Food and Drug Administration (FDA) for the treatment of external anogenital warts, actinic keratosis (AK), and superficial basal cell carcinoma (sBCC). The efficacy of imiquimod is primarily attributed to its ability to modulate both innate and adaptive immune responses, as well as its direct effects on cancer cells.

### 3.2. Immune Activation via Toll-like Receptors (TLRs)

Imiquimod exerts its immunomodulatory effects by activating Toll-like receptors 7 and 8 (TLR7/8) on various immune cells, including dendritic cells, macrophages, and natural killer (NK) cells. Upon binding to these receptors, imiquimod triggers the MyD88-dependent signaling pathway, leading to the activation of nuclear factor kappa B (NF-κB) and interferon regulatory factors (IRFs). This cascade leads to the production of pro-inflammatory cytokines, including interferon-alpha (IFN-α), tumor necrosis factor-alpha (TNF-α), interleukin-12 (IL-12), and interleukin-6 (IL-6). These cytokines enhance local inflammation, recruit additional immune cells to the tumor site, and stimulate antigen presentation, thereby promoting an anti-tumor immune response [8,9].

### 3.3. Stimulation of Adaptive Immunity

The cytokine milieu created by imiquimod also facilitates the activation and maturation of dendritic cells, which are crucial for the initiation of adaptive immune responses. Dendritic cells present tumor-associated antigens to naïve T cells, promoting their differentiation into cytotoxic CD8+ T cells and helper CD4+ T cells. These effector T cells then target and eliminate tumor cells. Furthermore, imiquimod enhances the activity of NK cells, which play a pivotal role in the early stages of tumor immunosurveillance [10].

### 3.4. Direct Pro-Apoptotic Effects on Tumor Cells

In addition to its immunomodulatory properties, imiquimod exhibits direct cytotoxic effects on tumor cells. It has been shown to induce endoplasmic reticulum (ER) stress in tumor cells, which activates intrinsic apoptotic pathways. This process involves the activation of p38 MAPK and NF-κB signaling, leading to the transcription of pro-apoptotic genes, such as those encoding Bax and Bak proteins. These proteins increase mitochondrial membrane permeability, promoting the release of cytochrome c and the subsequent activation of caspases. Concurrently, anti-apoptotic proteins, such as Bcl-2, are inhibited, further driving the apoptotic process [10].

### 3.5. Tumor-Selective Effects

Interestingly, imiquimod demonstrates tumor-selective cytotoxicity, sparing normal cells while inducing apoptosis in tumor cells. This selectivity may be attributed to the differential expression of TLR7/8 and the unique metabolic environment of tumor cells. Additionally, the local inflammatory response induced by imiquimod further amplifies its tumor-selective effects by creating a hostile microenvironment for cancer cells [9].

### 3.6. Clinical Implications and Outcomes

The multifaceted mechanisms of imiquimod make it an effective non-invasive treatment option for sBCC and AK. Clinical studies have demonstrated high clearance rates, minimal recurrence, and favorable cosmetic outcomes. However, the inflammatory response associated with imiquimod use can result in localized skin reactions, including erythema, edema, and crusting, which are generally self-limiting and indicative of its therapeutic activity [8].

### 3.7. Summary of Imiquimod Mechanisms of Action and Selection for Treatment

Imiquimod’s ability to modulate immune responses and directly induce apoptosis in tumor cells underpins its effectiveness in the treatment of sBCC and AK. Ongoing research continues to explore its potential applications in other malignancies and combination therapies to enhance its therapeutic efficacy.

In general, imiquimod is indicated for patients with small lesions, particularly in the treatment of external anogenital warts, actinic keratoses (AK), and superficial basal cell carcinomas (sBCCs).

## 4. Conventional Surgery

Conventional surgery is the most employed technique for removal of NMSCs. It is usually performed under local anesthesia in an ambulatory setting. Whenever it is possible to obtain wide margins (4 to 6 mm), cure rates may reach 90% to 95%. However, for tumors located on the face, scalp and ears, obtaining wide margins through conventional technique may lead to esthetic damage and sometimes functional impairment. Thus, the odds for complete removal of facial tumors drop considerably. For large malignant tumors of the face, head and neck, the chances of complete clearance of the tumor using conventional surgery are lower than 70% [11].

## 5. Mohs Micrographic Surgery (MMS)

As much as conventional surgery, MMS can be performed either at a physician’s office or at a hospital setting under local anesthesia, or sedation under general anesthesia (usually reserved for large tumors). For non-melanoma skin cancer, less invasive surgeries should be employed whenever feasible. Thus, most physicians prefer performing MMS under local anesthesia.

For surgeons, these tumors raise additional concerns. In regions close to the eyelids, nose, mouth, and ears, the surgeon’s removal of safety margins may necessitate the removal of large segments of skin from these sensitive areas. Therefore, for patients with facial cancer, it is necessary to spare as much healthy tissue as possible to preserve aesthetic and functional aspects. In this setting, MMS enables the narrowest possible margins. Regarding aesthetics, skin cancer located on the face causes greater concerns for patients.

MMS is the most precise surgical method for the treatment of non-melanoma skin cancer. This minimally invasive treatment technique for the treatment of NMSC was developed by the American surgeon Frederick Mohs and involves removing the lesion using a special technique, in which only small areas of surrounding skin and subcutaneous tissues are removed along with the skin cancer. Although the technique was initially developed by Mohs in the 1930s, it was modified and refined by him and others over the subsequent decades [12,13].

In MMS, the specimens removed are prepared through tissue staining and thin sections are performed by using a cryostat. The specimens are analyzed under a microscope located inside the operating room (or in a room close to where the patient is being operated on). Preparation of the specimens and the histologic analysis can be performed by the surgeon or dermatologist, or a pathologist [14].

### The Pathologist’s Role in Mohs Micrographic Surgery

The pathologist plays a critical role in MMS by ensuring accurate histological interpretation of excised tissue, guiding the surgeon in achieving complete tumor removal while preserving healthy tissue. Once the surgeon removes a layer of tissue, it is inked with multiple colors to denote orientation (e.g., medial, lateral, superior, and inferior edges). The tissue is carefully flattened and mapped to maintain precise spatial correlation between the specimen and its anatomical location on the patient [15]. Ensuring the tissue lies flat without distortion is essential to preserve an accurate representation of the tumor’s extent, as poorly oriented tissue can lead to false negatives or unnecessary excisions. The specimen is then rapidly frozen using a cryostat, a step crucial for maintaining tissue integrity while enabling rapid preparation of sections. Horizontal sections are made parallel to the tissue surface, contrasting with conventional pathology, where vertical sections are used, sampling only a fraction of the tissue margins. This technique allows the entire surgical margin, both peripheral and deep, to be examined on a single plane [16].

The tissue sections are subsequently stained, typically using hematoxylin and eosin (H&E), for routine histological examination. In some cases, toluidine blue or immunohistochemistry (IHC) may be used to highlight specific tumor features [17,18]. Proper slide preparation is essential to minimize artifacts, such as freezing artifacts or section folding, which could compromise diagnostic accuracy. The pathologist examines the entire surgical margin under a microscope, looking for residual tumor cells. For basal cell carcinoma (BCC), key histological features include basaloid cell nests, stromal retraction, and peripheral palisading [15]. For squamous cell carcinoma (SCC), keratin pearl formation and atypical squamous cells extending beyond the margins are the indicative features. Subtypes such as infiltrative or morpheaform BCC require scrutiny due to their subtle and diffuse invasion patterns [16]. Distinguishing residual tumor from benign adnexal structures or reactive atypia in fibrotic or inflamed tissue can be challenging and demands significant expertise.

Once the findings are documented, they are meticulously mapped onto the surgical diagram. Any positive margins are reported to the surgeon, specifying the exact anatomical location for further tissue removal. The pathologist must clearly communicate whether a residual tumor is present, and the type of margin involved (e.g., deep vs. lateral). All findings are carefully documented, including the histological characteristics of the tumor and margin status, and slides are preserved for potential review or consultation. This ensures quality control and allows for future re-evaluation if needed [19].

In addition to standard techniques, some centers incorporate advanced methods to enhance diagnostic precision. IHC is particularly useful in challenging cases, such as distinguishing poorly differentiated tumors from benign mimickers or identifying small foci of residual disease [18]. Emerging technologies, such as digital pathology and artificial intelligence (AI) also are being explored to identify subtle patterns in histological sections, potentially increasing diagnostic accuracy and efficiency [19]. The pathologist’s contribution to MMS is pivotal, as their expertise ensures the oncologic and aesthetic success of the procedure while maximizing tissue preservation.

Although some advocate that the availability of a pathologist for intraoperative analysis would increase the costs, this has not been confirmed in our practice. In our experience, analysis by a qualified skin pathologist has yielded excellent results. Although the surgeon needs to wait for a response by the pathologist to ensure that the margins are cancer free, the surgeon may shorten the operation by preparing the skin flap to close the defect. We have performed MMS with less than 30 min of operative time (mean of 45 min). Another advantage of having the MMS pathology analysis performed by a pathologist instead of the surgeon (or dermatologist) is that, for patients whose operation is performed under general anesthesia or sedation by an anesthesiologist (usually for patients with large tumors), additional skin lesions can be excised.

## 6. Local Reconstruction

If the analysis of the first specimen of MMS confirms that the entire margins have been achieved, the surgeon proceeds with skin closure: through direct wound closure or a skin flap or a graft. Otherwise, a new fragment of skin is removed (also using a special technique) for a new micrographic analysis. Therefore, MMS is only completed with the closure of the defect whenever the analysis shows that the cancer has been completely removed.

An important caveat in MMS is regarding the size of the margins. In MMS, not all tumor margins surrounding all tumors must be removed as very narrow. Very narrow margins are only used in the tumor areas located on the tumor side that are near noble structures of the face (especially the eyelids, periorbital areas, lips and nasal wings). For instance, whenever the tumor is located on the cheek (zygomatic area), only the upper margin (close to the eyelid) is obtained in the narrow fashion. In this case, a wider amount of skin may be removed with the lateral, medial and lower margins. Another example is the case of tumors located in the temporal areas of the face. In these instances, a narrow margin must be obtained in the tumor edge located close to the side corner of the eye to prevent deformities. In this setting, the surgeon may be more liberal by widening a little bit with the other margins. In other words, MMS does not necessarily demands that all margins surrounding the tumor are very narrow. As stated above, the use of a little wider margins on tumor sides located in less noble areas of the face considerably lowers the odds for positive margins that would demand additional histologic analysis. This has contributed to a much shorter operating time in MMS.

## 7. Mohs Micrographic Surgery for Basal Cell Cancer (BCC)

Using MMS method, it is not possible to measure the exact distance between the tumor and the margins. MMS only reveals whether the margins are tumor free or compromised by the tumor, whether the tumor is far or near the margins.

Although exceptions to this rule exist, as a category BCC is usually considered the least aggressive type of all types of skin cancer. Thus, BCCs usually demand the narrowest safety margins (2 to 5 mm). Thus, the most notable information for a pathology analysis of a BCC removal is whether the margins are cancer-free or not. Therefore, BCCs are considered the best tumors fitting into the MMS.

For primary BCC, MMS results in 98% to 99% chances of cure, in contrast to conventional surgery that yields 91% to 95% chances [20,21,22,23]. For recurrent BCC, MMS still enables 94% to 98% chances of cure, being substantially higher than those obtained with the use of conventional surgery (80% to 88%) [22,23,24].

A recent meta-analysis evaluated the efficacy of micrographic surgery versus conventional excision in reducing recurrence for basal cell carcinoma and squamous cell carcinoma [25]. For MMS, the overall recurrence rate was 3.1% vs. 5.3% for conventional surgery. In other words, there was a 52% reduction in recurrence of tumors treated with MMS as compared to conventional surgery.

## 8. Mohs Micrographic Surgery (MMS) for Other Types of Skin Cancer

Other than BCC, MMS is an important tool for the treatment of SCC. These tumors are more aggressive than BCCs, but their recurrent rates after MMS for SCCs are lower than those of MMS for BCCs [26]. Although for low-risk SCCs, MMS may provide cure rates comparable to BCCs, but for high-risk SCCs, recurrence rates are much higher. Marrazzo et al. have evaluated a total of 647 patients with high-risk SCC treated with MMS alone. The median follow-up was 36 months (range 12–192 months). The rate of local recurrence was 2.9%; local metastases occurred in 4.8% of the cohort; distant metastases occurred in 1.1% of the patients; and disease-specific deaths also occurred in an additional 1.1% of the patients. The authors concluded that various factors can be linked to poor outcomes in squamous cell carcinoma, especially poor differentiation and invasion beyond the subcutaneous fat can predict the risk of locoregional disease and disease-specific death. Mohs micrographic surgery alone provides excellent marginal control of high-risk squamous cell carcinoma and lower rates of measured poor outcomes. Also, there is a need to identify subsets of tumors benefiting from adjuvant prognostic testing (sentinel lymph node biopsy specimen, genetic expression profiling), with an aim to reduce morbidity and mortality of patients with high-risk SCCs.

Besides NMSCs, MMS has been employed for the treatment of Bowen disease of the skin (in situ SCCs) [27] and sometimes for actinic keratosis with high-grade dysplasia. Other benign tumors for which MMS has been employed include keratoacanthoma.

Apart from SCC and BCC, MMS has been employed for other malignant neoplasia are spindle cell carcinomas, sebaceous carcinomas, microcystic adnexal carcinomas, Merkel cell carcinoma, Paget’s disease of the breast, atypical fibroxanthoma, leiomyosarcoma and also dermatofibrosarcoma protuberans [28,29]. However, most of these tumors generally display much more aggressive behavior than BCCs. Thus, obtaining tumor free margins does not seem to be enough to provide the highest chances of cure. Therefore, MMS have been used for these tumors to prove that margins are free. Mostly, wide overall margins are obtained and analyzed through the Mohs method. Not only Mohs method is employed, but conventional pathology methods are also applied to estimate the size of the margins obtained. Whenever the margins are free but not wide enough to provide the highest possible chances of cure, a higher amount of surrounding skin is removed to widen the margins and potentially increase the odds for cure.

Although the performance of MMS for melanoma-in situ is more technically demanding, immuno-histochemical staining is feasible. Similarly to BCC and SCCs, MMS allows for 100% margin evaluation [30,31,32,33]. Recurrence rates using MMS for melanoma-in situ have been reported as ranging from 0 to 10%, being higher than those of wide margins [34]. The recommended margins for melanoma-in situ range from 5 mm to 10 mm [35,36,37,38]. A study provided that margins of 9 mm were able to provide a cure rate of 98.9%, being statistically superior to a 6 mm margin [30]. Several other studies support margins wider than 6 mm while performing MMS for melanoma [39,40,41,42,43,44].

## 9. Main Indications for Mohs Micrographic Surgery (MMS)

MMS is the gold-standard method for surgical treatment of NMSCs skin cancer, but MMS can be used for any skin cancer or premalignant condition to ascertain that the neoplasm has been completely removed [45]. For certain conditions, MMS is superior to conventional surgery. As for NMSCc of the eyelid and lip, MMS has higher cure rates than conventional surgery [46]. The main indications for MMS are:

- Location of the Tumor
  In situations where important structures from a functional or even aesthetic point of view are very close, making it difficult to use the safety margin concept:
  o Any tumor on the face (close to the eyelids, lacrimal caruncle, external auditory canal, nose, especially the free edge of the nasal wing, eyebrows, chin, lips, ears)
  o Forehead (scalp, temporal region)
  o Hands (including nail units)
  o Feet
  o Legs (region with higher chances of postoperative wound dehiscence)
  o Genitalia (perineal, perianal)
  o Nipples/areola, hands, feet, ankles, nail units
- Patient characteristics
  o Immunocompromised
  o Genetic syndromes related to skin cancer
  o Prior Radiation therapy
- Tumor Characteristics
  o Recurrent skin cancer
  o Tumor with poor clinical border delimitation.
  o BCCs with an infiltrative histological type, especially sclerodermiform carcinomas
  o Tumors with a diameter greater than 2 cm or that have been evolving for more than two years.

## 10. Management After Skin Cancer Removal Including Drugs to Prevent Flap and Skin Graft Failures

Whenever it is necessary to perform a reconstruction using skin flaps, the surgeon must be careful. Proper planning of a suitable skin flap involves thorough knowledge of local anatomy. It also includes patient-related factors such as patient age and facial anatomy. Patient age influences the elasticity of local skin and subcutaneous tissue. Intraoperatively, careful traction of the skin is mandatory. Proper choice of the sutures (absorbable versus non-absorbable) and the thickness of the suture is also important.

Postoperatively, antibiotics are employed prophylactically. The main antibiotics employed are Beta-lactam antibiotics. They are commonly recognized as first-line drugs of choice for treating cellulitis and erysipelas [47,48,49,50]. They are bactericidal antibiotics that inhibit cell wall biosynthesis by binding penicillin-binding proteins and thereby breaking the synthesis of peptidoglycan. Bacterial cell wall integrity depends on the synthesis of peptidoglycan. Specifically, cephalexin removes the D-alanine from the precursor of the peptidoglycan. Without proper cell wall synthesis, eventually bacteria will lyse. Amoxicillin (α-amino-*p*-hydroxybenzyl penicillin) is a semisynthetic derivative of penicillin with a structure similar to ampicillin but with better absorption when taken by mouth, thus yielding higher concentrations in blood and in urine. It inhibits cross-linkage between the linear peptidoglycan polymer chains that make up a major component of the bacterial cell wall.

Pentoxifylline, also known as oxpentifylline, is a xanthine derivative used as a drug to treat muscle pain in patients with symptomatic peripheral artery disease. This is its only FDA, MHRA and TGA-labeled indication [51]. Like other methylated xanthine derivatives, pentoxifylline is a competitive nonselective phosphodiesterase inhibitor [52] which raises intracellular cAMP, activates PKA, inhibits TNF [53,54] and leukotriene [55] synthesis, and reduces inflammation and innate immunity [55]. In addition, pentoxifylline improves red blood cell deformability (known as a hemorrhagic effect), reduces blood viscosity and decreases the potential for platelet aggregation and blood clot formation [56]. Pentoxifylline is also an antagonist at adenosine 2 receptors [57]. For postoperative cutaneous surgery, the off-label use of pentoxifylline is to promote vasodilation of the flap and to reduce viscosity in flap vessels.

## 11. Immunotherapy for Non-Melanoma Skin Cancer

The advent of immune checkpoint inhibitors, particularly anti-PD-1 therapies, has changed the therapeutic landscape for cutaneous squamous cell carcinoma, where until recently, it was primarily managed with surgery, radiation and cytotoxic chemotherapy.

Both anti-PD1 cemiplimab and pembrolizumab were initially approved for locally advanced and metastatic disease and have demonstrated significant clinical benefit, reaching high response rates. Both cemiplimab [57] and pembrolizumab [58] achieved objective response rates of 50% and 34%, respectively, with sustained disease control and improvements in quality of life (Table 1). Unlike traditional chemotherapy, they are very well tolerated, making them valuable for the older and comorbid population of SCC.

Considering the efficacy of anti-PD1 agents in advanced disease, cemiplimab was also explored in earlier stages of SCC. In a phase 2 [59] trial with 79 patients received cemiplimab for up to four doses before curative-intent surgery. Pathological complete response was achieved in 51% of patients, with an additional 13% achieving a pathological major response (≤10% viable tumor cells) (Table 1). These findings highlight the potential of neoadjuvant immunotherapy to reduce tumor burden, improve surgical outcomes, and limit the need for disfiguring resections, particularly in anatomically sensitive areas such as the head and neck.

Recently, cemiplimab was approved for use in the adjuvant setting for patients with high-risk SCC following surgery and radiation therapy. A phase 3 trial [60] of 415 patients with local or regional SCC, adjuvant cemiplimab given for a total of 48 weeks significantly improved disease-free survival compared to placebo (87.1% vs. 64.1% at 24 months), with substantial reduction in both locoregional (HR 0.20) and distant recurrence (HR 0.35) (Table 1). Cemiplimab has demonstrated clear benefit in both neoadjuvant and adjuvant settings. The consistent and durable responses across the different stages of the disease, combined with a favorable safety profile, position these therapies not as an alternative but as a new standard of care for select patients with cSCC.

PD-L1 is an adverse prognostic factor for other cancers, such as non-small cell lung cancer, pancreatic undifferentiated carcinoma, thyroid, and prostate cancer [62,63,64] and also for squamous cell carcinoma of the tongue and the floor of the mouth [64]. However, a recent meta-analysis revealed that there was a lack of prognostic significance of PD-L1 in oral squamous skin cancer [65].

Tumor mutational burden (TMB) is defined as the number of somatic mutations per megabase of interrogated genomic sequence and varies across malignancies. Panel sequencing-based estimates of TMB have largely replaced whole-exome sequencing-derived TMB in the clinic. Retrospective evidence suggests that TMB can predict the efficacy of immune checkpoint inhibitors, and data from KEYNOTE-158 led to the recent FDA approval pembrolizumab monotherapy for the subgroup of solid tumor patients with TMB ≥ 10 mut/Mb ICI alone or in combination has shown a benefit against chemotherapy only in a TMB high subgroup (defined as >243 mutations in exonic sequence or >10 mutation per Mb using FoundationOne assay) [66,67,68].

MB is also closely linked to other biomarkers, including microsatellite instability-high (MSI-H), which arises in a subset of human cancers due to deficient DNA mismatch repair (dMMR). Tumors with MSI-H/dMMR generally harbor elevated TMB, and MSI-H/dMMR is a well-established predictive biomarker for ICI responsiveness [69,70,71,72].

Expression of programmed death ligand-1 (PD-L1) on tumor cells and immune cells has become a widely used predictive biomarker for responsiveness to ICIs in several cancer types [73,74,75,76]. However, across many cancers, TMB and response to ICIs are independent of PD-L1 expression, underscoring the potential role of TMB as a complementary biomarker to identify additional patient subgroups likely to derive benefit from immunotherapy. However, TMB levels and response to immunotherapy in many cancers are independent of the level of PD-L1 expression, suggesting a potential role of TMB to identify additional subgroups of patients who may benefit from ICIs [77,78].

Promising new approaches include oncolytic viruses, which selectively replicate within tumor cells, leading to cell lysis, apoptosis, and release of viral progeny that propagate infection to neighboring cancer cells. Beyond direct cytotoxicity, these viruses can also stimulate antitumor immunity. Talimogene laherparepvec (T-VEC), an engineered HSV-1 modified to express GM-CSF and lacking ICP34.5 and ICP47, was the first oncolytic virus approved by the FDA for melanoma treatment [79].

Sonidegib and vismodegib are hedgehog pathway inhibitors that represent novel therapeutic options for basal cell carcinoma (BCC). BCC pathogenesis is frequently driven by aberrant activation of the hedgehog (Hh) signaling cascade, most often through mutations in the patched 1 (PTCH1) gene, which results in constitutive activation of smoothened (SMO) and downstream Hh signaling [80]. Since 2015, sonidegib has been approved for the treatment of locally advanced BCC in patient’s ineligible for curative surgery or radiotherapy [81]. This approval was supported by a randomized, double-blind, noncomparative trial in which 230 hedgehog inhibitor–naïve patients with locally advanced and/or metastatic BCC received sonidegib at daily doses of either 200 mg or 800 mg.

Vismodegib (Erivedge^®^) is the first-in-class oral small-molecule inhibitor of the Hedgehog (Hh) signaling pathway, whose abnormal activation is a key driver of basal cell carcinoma (BCC) [82,83]. In a recent clinical trial involving patients with orbital periocular BCC (opBCC), vismodegib demonstrated organ-preserving potential, with 67% of patients achieving a histologically confirmed complete response [84].

Neoadjuvant administration of vismodegib for periocular BCC, may reduce tumor size, facilitate resection, and reduce functional and aesthetic consequences of surgery. In a recent open label, noncomparative, multicenter, phase 2 study VISMONEO (NCT02667574), evaluated patients with ≥1 histologically confirmed facial BCC, inoperable or operable with functional or major aesthetic sequelae risk. Forty-four out of the total 56 patients (80%) experienced downstaging of the BCC. Of those, 27 patients (50% of the total study sample) achieved a complete response [85].

## 12. Mechanisms of Action of Radiotherapy in the Treatment of Basal Cell Carcinoma and Actinic Keratosis

Radiotherapy is a cornerstone in the treatment of various malignancies, including basal cell carcinoma (BCC) and actinic keratosis (AK). It relies on the use of ionizing radiation to induce lethal damage to cancer cells while sparing surrounding healthy tissue. The therapeutic effects of radiotherapy extend beyond the direct destruction of tumor cells, encompassing vascular disruption, modulation of the tumor microenvironment, and stimulation of anti-tumor immune responses.

### 12.1. Direct DNA Damage

The primary mechanism of radiotherapy involves direct and indirect damage to the DNA of cancer cells. Ionizing radiation induces single- and double-strand breaks in the DNA helix, leading to genomic instability and inhibiting the ability of tumor cells to replicate and survive. When the damage is irreparable, the affected cells undergo mitotic catastrophe, apoptosis, or senescence. The direct ionization of DNA by high-energy photons or charged particles plays a critical role in halting tumor proliferation and inducing cell death [86].

### 12.2. Indirect Effects Through Reactive Oxygen Species (ROS)

In addition to direct DNA damage, ionizing radiation generates reactive oxygen species (ROS) within cells. ROS cause oxidative damage to DNA, proteins, and lipids, exacerbating the cellular stress and contributing to tumor cell death. Tumor cells, which often exhibit impaired antioxidant defense, are particularly vulnerable to oxidative stress induced by radiotherapy [87].

### 12.3. Impact on Tumor Vasculature

Radiotherapy also affects the tumor vasculature by damaging endothelial cells and reducing angiogenesis. This effect limits the supply of oxygen and nutrients to the tumor, impairing its growth and survival. Moreover, vascular normalization induced by radiotherapy can enhance oxygenation within the tumor, thereby increasing its sensitivity to subsequent radiation doses [86].

### 12.4. Immunogenic Cell Death and Immune Activation

One of the most intriguing aspects of radiotherapy is its ability to stimulate an anti-tumor immune response through immunogenic cell death. Radiation-damaged cells release damage-associated molecular patterns (DAMPs), such as high-mobility group box 1 (HMGB1), adenosine triphosphate (ATP), and DNA fragments, into the tumor microenvironment. These molecules act as danger signals, activating dendritic cells and macrophages. The activation of these immune cells leads to the production of pro-inflammatory cytokines, including tumor necrosis factor-alpha (TNF-α), interleukin-1 beta (IL-1β), and interleukin-6 (IL-6), which amplify the immune response [87].

### 12.5. Antigen Presentation and T-Cell Activation

Following radiation-induced cell death, tumor antigens are released and subsequently captured by dendritic cells. These cells present the antigens to naïve T cells in regional lymph nodes, facilitating their differentiation into cytotoxic CD8+ T cells and helper CD4+ T cells. Activated T cells then migrate to the tumor site, where they recognize and destroy residual tumor cells through mechanisms such as perforin and granzyme release. This phenomenon, known as the abscopal effect, highlights the systemic immune-modulating potential of localized radiotherapy [88].

### 12.6. Macrophage-Mediated Clearance

Radiotherapy also enhances the phagocytic activity of macrophages, which are responsible for clearing dead tumor cells. The recruitment and activation of macrophages further contribute to the pro-inflammatory microenvironment, facilitating tumor clearance and potentially reducing the risk of recurrence [86].

### 12.7. Clinical Considerations

Radiotherapy is particularly effective for patients with BCC who are not suitable candidates for surgery or who have lesions in anatomically challenging locations. It also plays a role in treating extensive or recurrent AK lesions. Advances in radiotherapy techniques, such as intensity-modulated radiotherapy (IMRT) and stereotactic body radiotherapy (SBRT), have improved treatment precision, reducing collateral damage to surrounding healthy tissue while maintaining therapeutic efficacy [88].

Radiotherapy represents a multifaceted approach to treating BCC and AK, combining direct cytotoxic effects with immunomodulatory and anti-angiogenic mechanisms. Its ability to integrate localized tumor control with systemic immune activation underscores its role as a valuable treatment modality in dermatologic oncology. Ongoing research into combining radiotherapy with immunotherapy holds promise for enhancing its efficacy and expanding its indications.

Radiation therapy tumor control rates range from 90% to over 95% for primary tumors treated with radiation therapy alone [89]. For recurrent BCCs, the control rate ranges from 60% to 80%. Adjuvant radiation therapy may be recommended after surgical excision of high-risk BCCs to improve local control rates. The addition of radiation therapy to surgery has shown improved tumor control compared to surgery alone. Neoadjuvant radiation therapy refers to the administration of radiation therapy before surgery. It is considered for large, locally advanced BCCs to reduce the tumor and facilitate surgical resection [90,91].

Newer approaches in radiation therapy have become available recently. Proton therapy and immunoradiotherapies are promising treatments that have been actively employed. PT is a specialized form of external beam radiation therapy that utilizes protons, rather than conventional X-ray photons, to deliver radiation to the tumor site. As compared to photon-based treatments, protons have unique physical properties that allow for precise dose deposition, resulting in superior dose conformity and sparing of healthy tissues. Proton therapy may be considered for BCCs in challenging locations, complex geometries, or tumors in proximity to critical structures [89,92]. The combination of radiation therapy and systemic therapies, such as targeted therapies or immunotherapy may increase treatment efficacy in patients with advanced or metastatic SCC and BCC cases.

## Figures and Tables

**Table 1:** Summary of Clinical Studies Involving Immunotherapy for Non-Melanoma Skin Cancer.

Study	N pts	Squamous Cell Carcinoma	Design	Regimen	Objective Response Rate (95% CI)	Response to Treatment
Migden MR et al., 2018 [58]	28/59 pts	Advanced disease	Phase I/II Single Arm Study	Cemiplimab	Phase I: 50% (30–70) Phase II: 47% (34–61)	(47–50% response)
Grob JJ et al., 2020 [59]	105 pts	Advanced disease	Phase II Single Arm Study	Pembrolizumab	34.3% (25.3–44.2)	Median Progression Free Survival 6.9 m (3.1–8.5 m)
Gross ND et al., 2022 [60]	79 pts.	Neoadjuvant	Phase II Single Arm Study	Cemiplimab	68% (57–78) 51% pathological complete response	
Rischin D et al., 2025 [61]	415 pts.	Adjuvant	Phase III Randomized trial	Cemiplimab vs. Placebo	---	Relapse Free Survival 87.1% (80.3–91.6) vs. 64.1% (55.9–71.1) HR 0.32 *p* < 0.001

## Data Availability

No new data were created or analyzed in this study.

## Abbreviations

TNF-α	Tumor necrosis factor-alpha
Ak	Actinic keratosis
BCC	Basal cell carcinomas
ER	Endoplasmic reticulum (ER)
FDA	Food and Drug Administration (FDA)
IFN-α	Interferon-alpha
IL-12	Interleukin-12
IL-6	Interleukin-6
IRFs	Interferon regulatory factors
MMS	Mohs Micrographic Surgery (MMS)
MYD88	Myeloid differentiation primary response 88
MyD88	Myeloid differentiation primary response gene 88
NF-κB	Nuclear factor kappa B
NK	Natural killer
p38 MAPK	p38 mitogen-activated protein kinases
sBCC	Superficial basal cell carcinoma
SCC	Squamous cell carcinoma
TLR7/8	Toll-like receptors 7 and 8
